# Genome-Wide Identification of NF-YA Transcription Factors in Strawberry and Their Responses to Salt Stress

**DOI:** 10.3390/plants15101475

**Published:** 2026-05-12

**Authors:** Jiajie Yu, Xiang Zhang, Shuang Wang, Dian Wang, Yingzhu Gao, Xiaohong Li

**Affiliations:** School of Agriculture, Liaodong University, Dandong 118003, China; yujiajie@liaodongu.edu.cn (J.Y.); zhangxiang@liaodongu.edu.cn (X.Z.); ws15542199225@163.com (S.W.); 18841533141@163.com (D.W.); gyingzhu@163.com (Y.G.)

**Keywords:** NF-YA transcription factor, strawberry (*Fragaria* × *ananassa*), genome-wide identification, expression analysis, salt stress

## Abstract

Nuclear Factor Y (NF-Y) transcription factor family plays essential roles in plant growth, development, and abiotic stress responses. However, the NF-YA subfamily in cultivated strawberry (*Fragaria* × *ananassa*) has not been systematically characterized from a genome-wide range. In this study, 27 *FaNF-YA* genes were identified from the octoploid strawberry genome and classified into four phylogenetic groups. With bioinformatic methods, it was found that all FaNF-YA proteins contain a highly conserved CCAAT-binding domain, while their exon–intron structures and motif compositions vary among groups. Promoter *cis*-acting element analysis revealed various stress- and hormone-responsive motifs, including ABRE, MYB, MYC, and MeJA-responsive elements. With molecular biology methods, organ-specific expression profiling was generated and showed that *FaNF-YA* genes exhibit distinct spatial expression patterns, with extremely low transcript abundance in fruit. Under salt stress, several *FaNF-YA* groups (e.g., *FaNF-YA14/16/18/22*) were dramatically induced, which indicated their potential involvement in salt tolerance. Heterologous expression of FaNF-YA7 and FaNF-YA9 in yeast enhanced salt tolerance, and these two proteins did not exhibit transcription-activating activity in the yeast GAL4 system. This study provides a reference for understanding the roles of *NF-YA* genes in responses to abiotic stresses and potential targets for molecular breeding of stress-tolerant strawberry cultivars.

## 1. Introduction

Global agricultural productivity is increasingly constrained by adverse environmental conditions, including drought, soil salinization, and extreme temperature fluctuations. These abiotic stresses impose significant limitations on plant growth, development, and reproductive success, which collectively threaten food security in an era of climate change. Sessile to environmental stresses, plants have evolved comprehensive molecular mechanisms to perceive stress signals and initiate adaptive responses through dynamic reprogramming of gene expression. Elucidating the transcriptional regulatory networks underlying stress adaptation is therefore essential for enhancing the stress tolerance of plants through molecular breeding or genetic engineering approaches.

Transcription factors have been proven to play key roles in the molecular responses of plants upon abiotic stresses [1,2]. The Nuclear Factor Y (NF-Y) transcription factor, also named Heme Activator Protein (HAP) or CCAAT-binding factor (CBF), functions in the form of a heterotrimeric complex, which exists widely across eukaryotic lineages [3,4]. The NF-Y complex is assembled by three distinct subunits, NF-YA, NF-YB, and NF-YC, in a strictly ordered manner [5]. The NF-YB and NF-YC subunits initially form a dimer in the cytoplasm prior to nuclear translocation, whereupon the NF-YA subunit is recruited to constitute the competent heterotrimer [6]. This functional complex exhibits sequence-specific recognition of the CCAAT box [3,7,8]. The NF-YA subunit is functionally indispensable, harboring both the DNA-binding and interaction domains necessary for binding to the NF-YB/YC heterodimer [9]. Between plants and other organisms exists an evolutionary divergence: compared to single copies of each subunit in other organisms, there are expanded family members of NF-YA, NF-YB, and NF-YC, which suggests extensive combinatorial assembly potential and functional diversification [8].

NF-Y transcription factors have been proven by numerous studies to be widely involved in various biological processes of plant growth and development, including embryogenesis, gamete development, seed germination, root morphogenesis, flowering, fruiting, hypocotyl elongation, photosynthesis, starch biosynthesis, and abscisic acid (ABA) signaling [8,10,11,12,13,14,15,16,17,18,19,20,21]. Functional characterization of *NF-YA* genes across multiple plant species has revealed their wide aspects of involvement in both developmental processes and stress responses. In *Arabidopsis thaliana*, multiple NF-YA genes were detected to respond to drought, salinity, and nutrient limitation, with expression fine-tuned through both transcriptional control and post-transcriptional regulation mediated by the microRNA169 (miR169) family [22]. The overexpression of *NF-YA2*, *NF-YA7*, or *NF-YA10* conferred enhanced tolerance to multiple abiotic stressors. In rice (*Oryza sativa*), overexpression of *OsNF-YA7* enhanced drought tolerance by mediating an abscisic acid-independent pathway [23]. It demonstrated functional conservation of stress regulatory mechanisms across monocot and dicot species. In soybean (*Glycine max*), *GmNFYA5* exhibited the highest upregulation in expression level upon drought stress among all the twenty-one NF-YA family members [24]. Overexpression of *GmNFYA5* in both transgenic *Arabidopsis* and soybean improved drought tolerance by reducing stomatal aperture and thereby water loss. In sorghum (*Sorghum bicolor*), eight of the nine identified *SbNF-YA* genes showed differential expression under drought stress [25]. The overexpression of *SbNF-YA6* in *Arabidopsis* enhanced the drought tolerance of the transgenic plants, with transgenic lines exhibiting increased germination rates, longer roots, higher fresh weight, elevated activities of antioxidant enzymes, and increased accumulation of osmoprotectants. In wheat (*Triticum aestivum*), expression profiling of *TaNF-YA* genes revealed differential regulation under drought, waterlogging, and nitrogen limitation conditions, as well as in response to ABA and 1-aminocyclopropane-1-carboxylic acid treatments [26]. In grape (*Vitis amurensis*), *VaNF-YA6* was proven to play positive roles in salt and drought tolerance [27]. Its overexpression in grapevine leaves and *Arabidopsis* significantly enhanced tolerance to both stresses by improving the expression levels of *VvSOS2*, *VvSOS3*, *VvABF3*, and *VvCPK6*, as well as increasing enzyme activities and protective substances. Collectively, these multi-species investigations have established NF-YA transcription factors as conserved yet functionally diversified regulators that integrate developmental programs with abiotic stress responses.

The extensive genome-wide characterizations of NF-YA gene family members across diverse angiosperms reveal substantial variation in family size. A total of eight *NF-YA* genes were identified in jujube [28], as well as 20 in cabbage [29], 12 in pecan [30], 11 in potato [31], 23 in tobacco [32], 12 in durum wheat [33], seven in finger millet [34], 12 in melon [35], 13 in poplar [36], nine in sugarcane [37], and 17 in switchgrass [38], whereas a comprehensive inventory and systematic analysis of *NF-YA* genes in cultivated strawberry (*Fragaria* × *ananassa*) remain conspicuously lacking. This knowledge gap is particularly significant given the global economic importance of strawberry as a high-value fruit crop and its recognized susceptibility to multiple abiotic stresses, including drought, salinity, and extreme temperatures, which severely constrain yield and fruit quality under field conditions [39,40,41,42,43]. The octoploid nature of cultivated strawberry, coupled with its complex domestication history and the availability of high-quality reference genomes, provides a reliable resource to investigate the evolutionary dynamics and functional diversification of the NF-YA family following polyploidization. Furthermore, the documented roles of NF-YA transcription factors in regulating stress-responsive gene expression, developmental transitions, and phytohormone signaling pathways across multiple plant species provide compelling evidence that strawberry NF-YA homologs may similarly participate in these critical processes.

## 2. Results

### 2.1. Identification and Characteristics of NF-YAs in Strawberry

**Figure 1:**
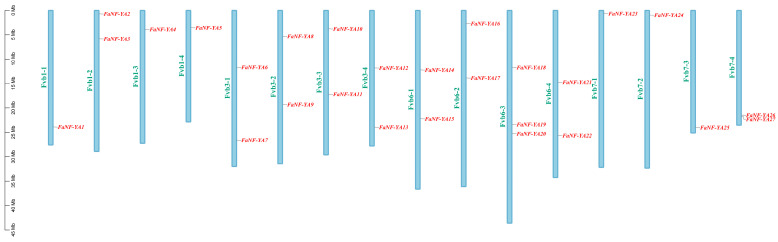
Chromosome location of *FaNF-YA*s. Blue square represents strawberry chromosome (chromosome numbers in green font). The scale bar is positioned to the left of the heatmap to measure the length of chromosome.

To comprehensively identify NF-YA family members in the cultivated strawberry (*Fragaria* × *ananassa*) genome, a systematic BLASTP search was performed using the Hidden Markov Model (HMM) profile of the NF-YA conserved domain retrieved from the Pfam database, using *Arabidopsis thaliana* NF-YA protein sequences as query sequences. After removal of redundant and incomplete sequences, a total of 27 *FaNF-YA* genes were identified. These genes were designated as *FaNF-YA1* to *FaNF-YA27* based on their distributed location on strawberry chromosomes (Figure 1).

By using the ExPASy ProtParam tool, the physiochemical properties of the 27 FaNF-YA proteins were investigated, including molecular weights (MWs), theoretical isoelectric points (pIs), amino acid lengths, instability indices, aliphatic indices, and grand average of hydropathicity (GRAVY) values (Table 1). These proteins exhibited substantial variation in length, ranging from 145 amino acids (FaNF-YA27) to 424 amino acids (FaNF-YA5). Correspondingly, the molecular weights of these proteins varied from 15.87 kDa (FaNF-YA27) to 46.99 kDa (FaNF-YA5). The theoretical isoelectric points (pIs) ranged from 6.79 (FaNF-YA7, FaNF-YA8, FaNF-YA13) to 10.13 (FaNF-YA6), which indicated that the majority of FaNF-YA proteins were basic in nature. Instability index analysis revealed that 25 out of 27 FaNF-YA proteins exhibited instability indices exceeding 40, which classified them as unstable proteins, with FaNF-YA27 (instability index = 26.82) and FaNF-YA25 (37.08) being the only exceptions. The aliphatic index, which serves as an indicator of thermostability, ranged from 49.97 (FaNF-YA9, FaNF-YA11) to 72.85 (FaNF-YA5), which suggested variable thermostability among family members. Furthermore, all 27 FaNF-YA proteins displayed negative GRAVY values, ranging from −1.084 (FaNF-YA6) to −0.411 (FaNF-YA5), which indicated their hydrophilic nature.

### 2.2. Conserved Domains and Phylogenetic Relationships of FaNF-YAs

**Figure 2:**
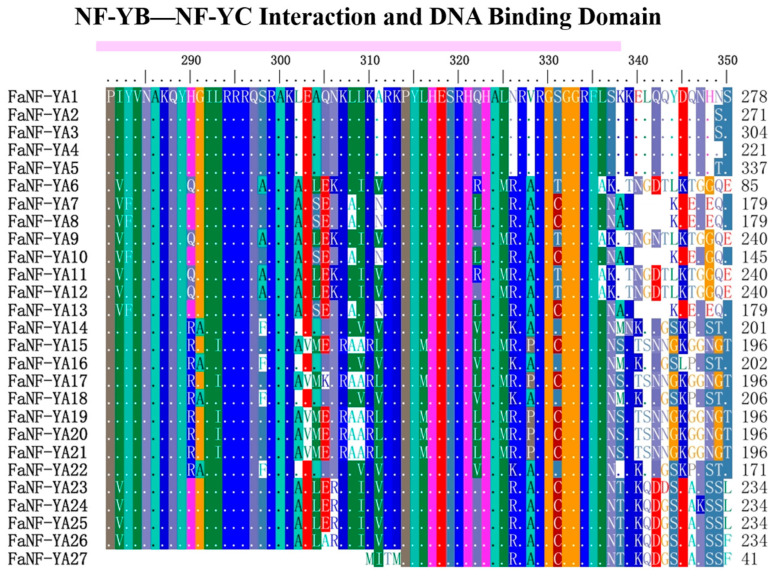
Multiple sequence alignment of FaNF-YA members. Amplified colored letters above protein sequences indicate a motif. Different-colored letters represent different amino acids. Pink line indicates the range of NF-YB—NF-YC Interaction and DNA Binding Domain.

To characterize the structural conservation of the 27 FaNF-YA proteins, multiple sequence alignment was performed using TBtools (v2.441). As shown in Figure 2, each FaNF-YA protein contained one NF-YA–NF-YC interaction domain and one DNA binding domain, which was a hallmark of plant NF-YA transcription factors. Outside the core conserved region, the N-terminal and C-terminal sequences exhibit considerable length and amino acid variability.

**Figure 3:**
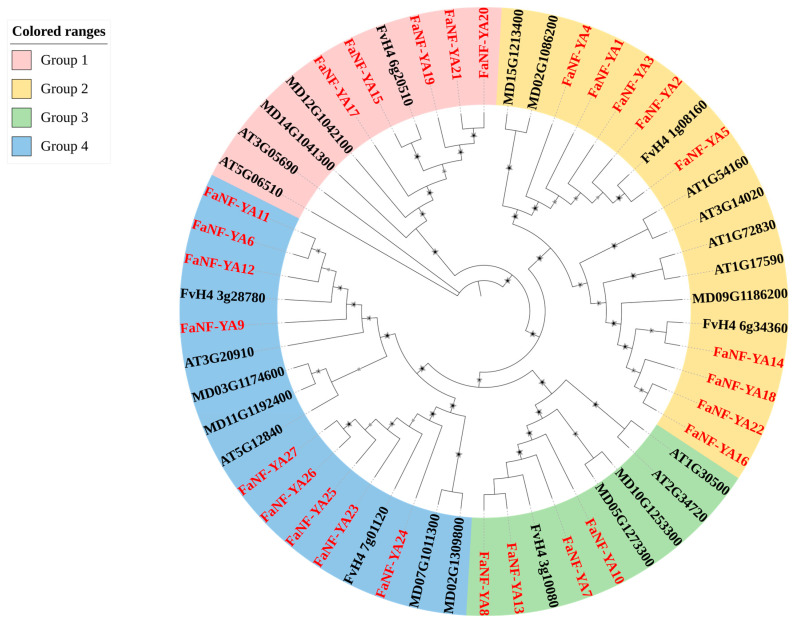
Phylogenetic relationships of NF-YA members in *Arabidopsis* (*Arabidopsis thaliana*), apple (*Malus domestica*), woodland strawberry (*Fragaria vesca*), and cultivated strawberry (*Fragaria* × *ananassa*). Stars represent bootstraps.

To elucidate the evolutionary relationships among the 27 FaNF-YA proteins identified in cultivated strawberry (*Fragaria* × *ananassa*), a phylogenetic tree was constructed with the neighbor-joining (NJ) method based on the full-length amino acid sequences of NF-YA proteins from *Arabidopsis* (*Arabidopsis thaliana*), apple (*Malus domestica*), woodland strawberry (*Fragaria vesca*), and cultivated strawberry (*Fragaria* × *ananassa*). As shown in Figure 3, all NF-YA proteins were clearly divided into four major groups (Groups 1–4). There were five FaNF-YA members (FaNF-YA15, -17, -19, -20 and -21) in Group 1, nine (FaNF-YA1, -2, -3, -4, -5, -14, -16, -18 and -22) in Group 2, four (FaNF-YA7, -8, 10 and -13) in Group 3, and nine (FaNF-YA6, -9, -11, -12, -23, -24, -25, -26, -27) in Group 4. FaNF-YA members were predominantly clustered together with their orthologs from woodland strawberry.

### 2.3. Gene Structures and Conserved Motifs of FaNF-YAs

**Figure 4:**
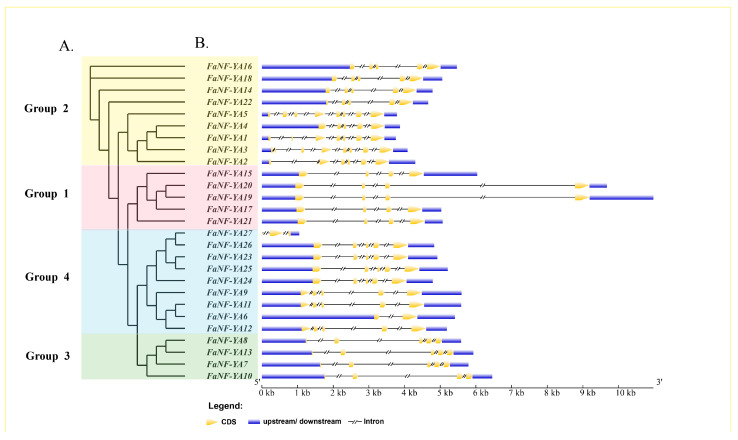
Gene structures of *FaNF-YA*s. (**A**) Phylogenetic tree constructed based on the full-length sequences of the FaNF-YAs. (**B**) Exon–intron structures of the *FaNF-YA*s.

To investigate the gene structures (exon–intron organizations) of the *FaNF-YA* genes, the coding sequences (CDSs) and their corresponding genomic sequences were submitted to the GSDS (Gene Structure Display Server) for comparative analysis. As illustrated in Figure 4, the gene structures of *FaNF-YA* members varied considerably in terms of intron number. However, when grouped according to the phylogenetic tree (Groups 1–4), clear structural conservation was observed within each group. In Group 1, all of the *FaNF-YA*s had three introns. In Group 2, 5/9 of the *FaNF-YA*s had four introns, while the other four members had six or seven introns. In Group 3, all of the *FaNF-YA*s had three or four introns. In Group 4, most (7/9) of the *FaNF-YA*s had four introns, while the other two members had only one or two introns.

**Figure 5:**
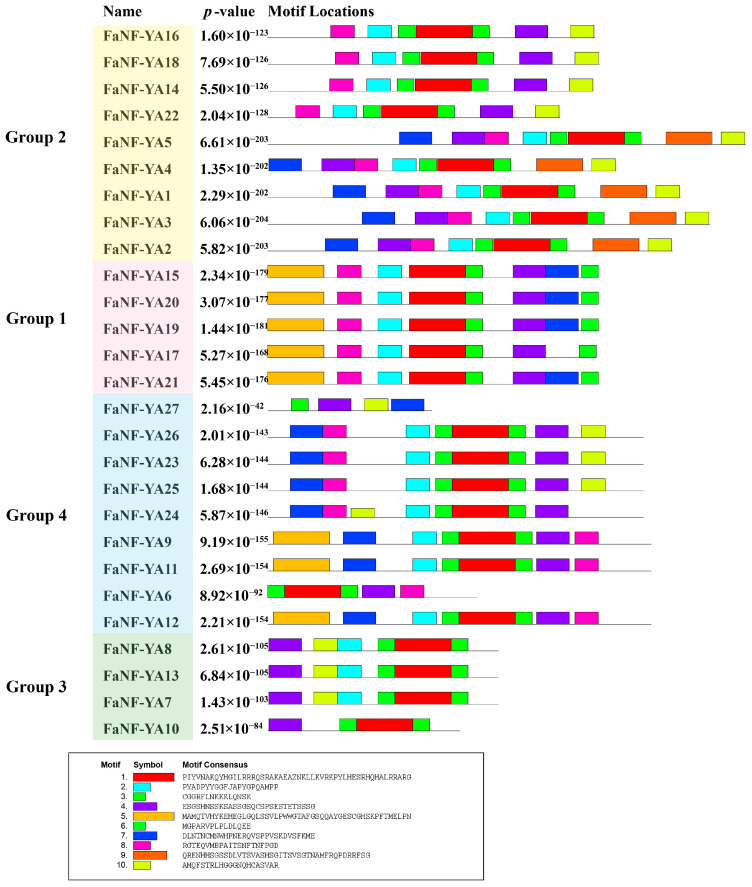
Motif distribution in FaNF-YAs.

To identify conserved amino acid sequence patterns among the 27 FaNF-YA proteins, the MEME suite was employed. A total of ten distinct motifs were identified, and the distribution of these motifs across the FaNF-YA members is presented in Figure 5. Overall, the motif distribution pattern was largely consistent with the phylogenetic groupings. Notably, Motif 3 and Motif 4 were found in all the FaNF-YAs. Motif 1 and Motif 6 were consistently located in close proximity to each other.

### 2.4. Intraspecies Collinearity of FaNF-YAs

**Figure 6:**
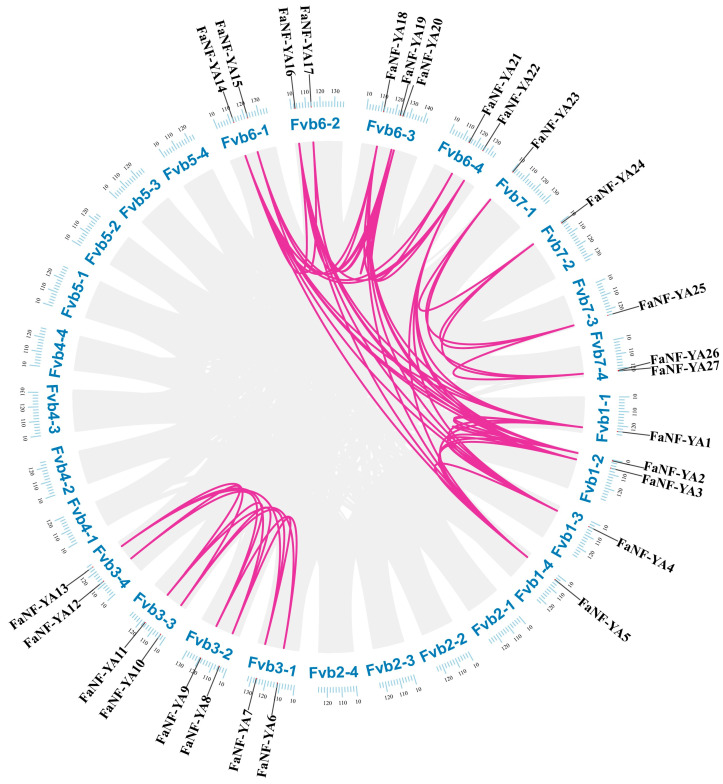
Intraspecies collinear relationships between *FaNF-YA*s. Pink lines indicate collinear relationships, and grey lines indicate all of the collinear backgrounds in the cultivated strawberry genome.

To investigate the expansion mechanisms and evolutionary constraints acting on the FaNF-YA gene family, intraspecies collinearity analysis was performed between *FaNF-YA* genes. As shown in Figure 6, a total of 62 collinear gene pairs were identified between *FaNF-YA*s.

Ka/Ks (non-synonymous substitution/synonymous substitution) ratios were calculated for each duplicated pair to infer the type of selection pressure (Table 2). The selective pressure acting on duplicated gene pairs was inferred from Ka/Ks ratios: purifying selection when <1, neutral evolution when =1, and positive selection when >1. As summarized in Table 2, the majority of duplicated *FaNF-YA* pairs exhibited Ka/Ks values below 1, ranging from 0.0896 (*FaNF-YA7*/*FaNF-YA13*) to 0.9916 (*FaNF-YA23*/*FaNF-YA24*).

### 2.5. Interspecies Collinearity of FaNF-YAs

**Figure 7:**
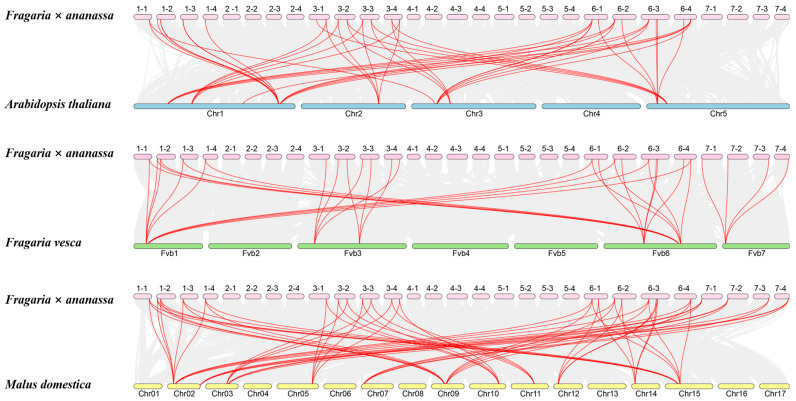
Interspecies collinear relationships between *NF-YA* genes from cultivated strawberry and *Arabidopsis*, cultivated strawberry and diploid strawberry, cultivated strawberry and apple. Red lines denote collinear *NF-YA* gene pairs, gray lines represent background collinear blocks, and chromosome numbers are indicated adjacent to each chromosome.

To investigate the evolutionary conservation of *NF-YA* genes across different plant species, comparative collinearity analyses were performed between *FaNF-YA*s and *AtNF-Ya*s; *FaNF-YA*s and *FvNF-Ya*s; and *FaNF-YA*s and *MdNF-YA*s. Collinear relationships were identified using MCScanX and visualized as syntenic maps (Figure 7). The detailed orthologous gene pairs are listed in Supplementary Tables S1–S3. A total of 39, 34 and 60 collinear gene pairs were identified between *FaNF-YA*s and *AtNF-Ya*s; *FaNF-YA*s and *FvNF-Ya*s; and *FaNF-YA*s and *MdNF-YA*s, respectively. Several *FaNF-YA* genes were collinear to more than one ortholog in certain species. For instance, *FaNF-YA1*, *-2*, *-3*, *-4*, *-5*, *-14*, *-16*, *-18*, *-22* were collinear with two distinct *FvNF-YA* genes. *FaNF-YA1*, *-2*, *-3*, *-4*, *-5* were collinear with three distinct *MdNF-YA* genes. Additionally, there was a circumstance where many *FaNF-YA* genes were simultaneously collinear with certain orthologs from other species. For instance, *FaNF-YA1*, *-2*, *-3*, *-4*, *-5*, *-14*, *-16*, *-18*, *-22* were collinear with AT1G72830.2 AtNF-YA1 (AT1G72830.2) and AtNF-YA3 (AT3G14020.1).

### 2.6. cis-Acting Elements in the Promoters of FaNF-YAs

**Figure 8:**
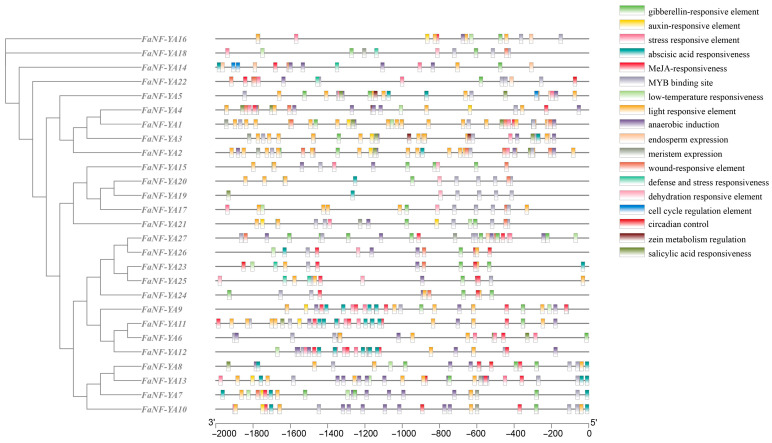
*cis*-acting elements in the promoters of *FaNF-YA* genes. Different-colored boxes symbolize different *cis*-acting elements. Scale bar measures the length of promoters.

Promoter analysis (2000 bp upstream region) of all 27 *FaNF-YA* genes using PlantCARE revealed 18 distinct *cis*-acting regulatory elements. They were classified into four categories: light-responsive, phytohormone-responsive, stress-responsive, and growth/development-related (Figure 8, Supplementary Table S4a,b). Light-responsive elements were the most abundant, with 130 occurrences across all promoters. Stress-related elements were also highly represented. MYB binding sites (91 occurrences) and anaerobic induction elements (72 occurrences) were particularly prevalent. In addition, stress-responsive elements (31 occurrences), wound-responsive elements (24 occurrences), low-temperature responsiveness elements (15 occurrences), dehydration-responsive elements (10 occurrences), and defense and stress-responsiveness elements (six occurrences) were detected.

Notably, the distribution of these *cis*-acting elements varied among individual *FaNF-YA* promoters. For instance, *FaNF-YA1*, *FaNF-YA2*, *FaNF-YA3*, and *FaNF-YA9* contained the highest numbers of ABA-responsive elements, while *FaNF-YA7*, *FaNF-YA8*, and *FaNF-YA13* were enriched in MeJA-responsive and MYB binding sites. In contrast, *FaNF-YA23* through *FaNF-YA27* displayed a higher proportion of light-responsive and circadian control elements.

### 2.7. Organ-Specific Expression Profiles of FaNF-YAs

**Figure 9:**
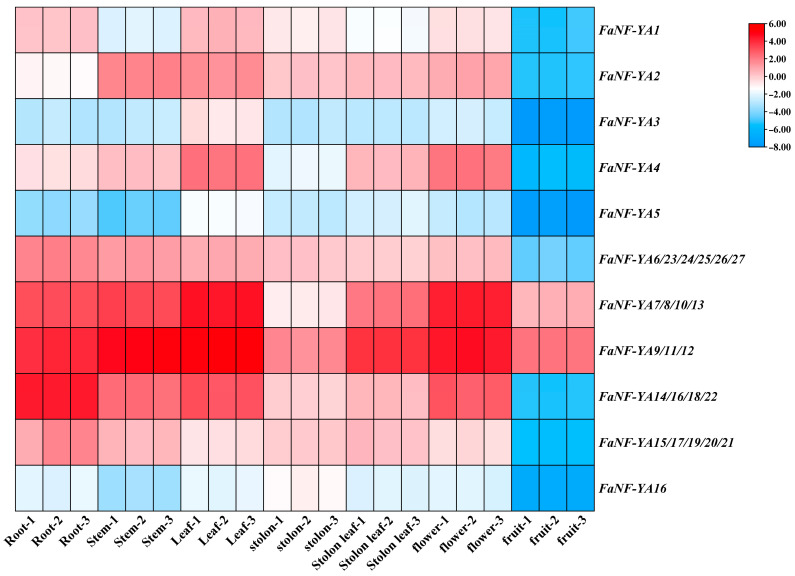
Organ-specific expression patterns of *FaNF-YA* genes. qRT-PCR was performed with *FaActin* as the internal reference gene. Relative expression levels were calculated using the 2^−ΔΔCt^ method, and log_2_-transformed values (−ΔΔCt) were used to generate the heatmap. The color scale is shown on the right.

To investigate the spatial expression patterns of *FaNF-YA* genes, qRT-PCR was performed on seven distinct organs of cultivated strawberry: root, stem, leaf, stolon, stolon leaf, flower, and fruit (Figure 9). Individual *FaNF-YA* genes showed distinct organ preference patterns. For instance, *FaNF-YA1* was expressed at comparable levels in root and leaf, but its transcript abundance in fruit was only about 0.02-fold of that in root. *FaNF-YA2* exhibited the highest expression in stem, with levels approximately 8-fold higher than in root, followed by leaf (approximately 7-fold), flower (approximately 4-fold), and stolon leaf (approximately 1.2-fold). *FaNF-YA4* was most abundant in leaf (approximately 7-fold of root) and flower (approximately 6-fold of root), whereas its expression in stolon was only about 0.4-fold of root. All examined *FaNF-YA* genes or groups exhibited extremely low expression in fruit, with fruit/root ratios ranging from 0.01 to 0.04. The specific relative qRT-PCR data are listed in Supplementary Table S5.

### 2.8. Expression Profiles of FaNF-YAs upon Salt Stress

**Figure 10:**
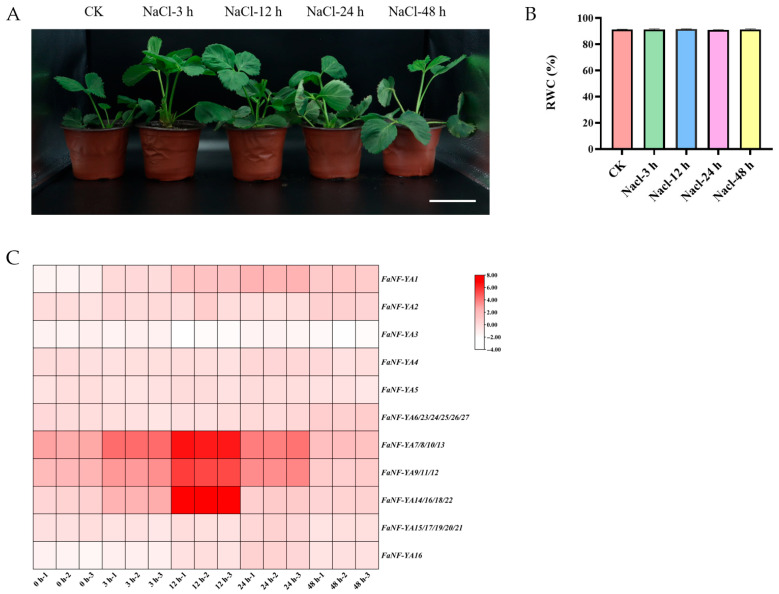
Phenotypic and expressional responses of *FaNF-YA* genes upon salt stress. (**A**) Phenotype of strawberry seedlings under salt stress. Experimental groups were strawberry plants exposed to 200 mM NaCl for 0 h (control, no treatments), 3 h, 12 h, 24 h, and 48 h. Three biological replicates were used. Scale bar = 10 cm. (**B**) Relative water content (RWC) of strawberry leaves under salt stress. RWC was measured at each time point of salt stress treatment. Three biological replicates were used. No significant differences in RWC were observed among the different treatment durations (*p* > 0.05, one-way ANOVA). (**C**) Relative expression levels of *FaNF-YA* genes under salt stress. Plant samples were harvested at 0 h (untreated control), 3 h, 12 h, 24 h, and 48 h following 200 mM NaCl exposure. qRT-PCR data (normalized by using *FaActin* as internal reference gene) were processed via the 2^−ΔΔCt^ method, and log_2_-transformed fold changes (−ΔΔCt) are presented as a heatmap. The color scale is shown on the right.

To investigate the potential involvement of *FaNF-YA* genes in salt (NaCl) stress responses, the transcript levels of individual *FaNF-YA* genes and pooled gene groups were examined by qRT-PCR in strawberry seedlings treated with NaCl for 0, 3, 12, 24, and 48 h. As shown in Figure 10A,B, the salt treatment for up to 48 h did not cause visible wilting, chlorosis, or necrosis in the strawberry plants, and there was no significant difference in RWC between the salt-treated plants and the control (0 h). Salt stress induced widespread upregulation of *FaNF-YA* genes, but with markedly different magnitudes and temporal patterns. *FaNF-YA1* showed a progressive increase in transcript abundance over time: expression was upregulated approximately 3-fold at 3 h, 7-fold at 12 h, 14-fold at 24 h, and 5-fold at 48 h relative to 0 h, indicating a strong and sustained induction by salt stress. *FaNF-YA2* was moderately induced, with peak expression at 3 h (approximately 1.3-fold) and 12 h (approximately 1.7-fold), followed by a gradual decline. *FaNF-YA3* showed little change across all time points, with fold changes ranging from 0.6 to 1.1, which suggested that this gene was not responsive to salt stress. *FaNF-YA4* was induced approximately 2-fold at 3 h and 12 h, with a slight increase to approximately 3-fold at 24 h, before returning to basal levels at 48 h. *FaNF-YA5* displayed a modest but consistent induction (approximately 2-fold) from 3 h to 24 h, with a slight reduction at 48 h. *FaNF-YA16* was also induced, with peak expression at 24 h (approximately 3-fold) and sustained upregulation at 12 h (approximately 2-fold) and 48 h (approximately 2-fold).

The group *FaNF-YA7/8/10/13* was dramatically induced by salt stress: expression increased from 0 h to approximately 55-fold at 3 h, peaked at 12 h with an extraordinary 230-fold increase, then declined to 40-fold at 24 h and 9-fold at 48 h. The group *FaNF-YA9/11/12* also showed strong induction, with fold changes of approximately 25-fold at 3 h, 110-fold at 12 h, 30-fold at 24 h, and 5-fold at 48 h. The group *FaNF-YA14/16/18/22* exhibited a similar pattern, with moderate induction at 3 h (approximately 4-fold), a dramatic peak at 12 h (approximately 360-fold), followed by declines to 5-fold at 24 h and 3.5-fold at 48 h. The specific relative qRT-PCR data are listed in Supplementary Table S6.

### 2.9. Transcription-Activating Activity of FaNF-YA7 and FaNF-YA9

**Figure 11:**
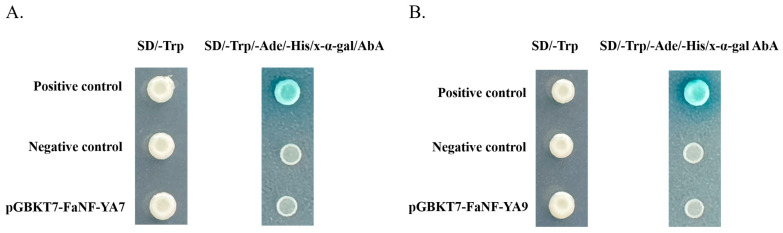
Transcription-activating activity of FaNF-YA7 and FaNF-YA9. (**A**) Detection of the transcription-activating activity of FaNF-YA7. (**B**) Detection of the transcription-activating activity of FaNF-YA9. Yeast transformants were grown on SD/-Trp (selection for pGBKT7) and SD/-Trp/-Ade/-His supplemented with 500 ng/L AbA (reporter activation selection). The positive control was pGBKT-53 + pGADT7-T; the negative control was empty pGBKT7.

To determine the transcription-activating activity of FaNF-YAs, FaNF-YA7 and FaNF-YA9, which are responsive to salt stress, were subjected to yeast transformation analysis. These two proteins were introduced into yeast cells, respectively, after being integrated into the pGBKT7 vector. As shown in Figure 11A, both pGBKT7-FaNF-YA7 and pGBKT7-FaNF-YA9 transformants, together with the positive control (pGBKT7-53 + pGADT7-T) and the negative control (empty pGBKT7), grew well on SD/-Trp, which indicated successful transformation. In contrast (Figure 11B), pGBKT7-FaNF-YA7 and pGBKT7-FaNF-YA9 transformants, as well as the negative control, failed to grow on SD/-Trp/-Ade/-His/x-α-gal medium, while the positive control grew vigorously and developed blue coloration on this medium. This showed that FaNF-YA7 and FaNF-YA9 lacked transcription-activating activity under the tested yeast system.

### 2.10. Enhanced Salt Tolerance by FaNF-YA7 and FaNF-YA9 in Yeast

**Figure 12:**
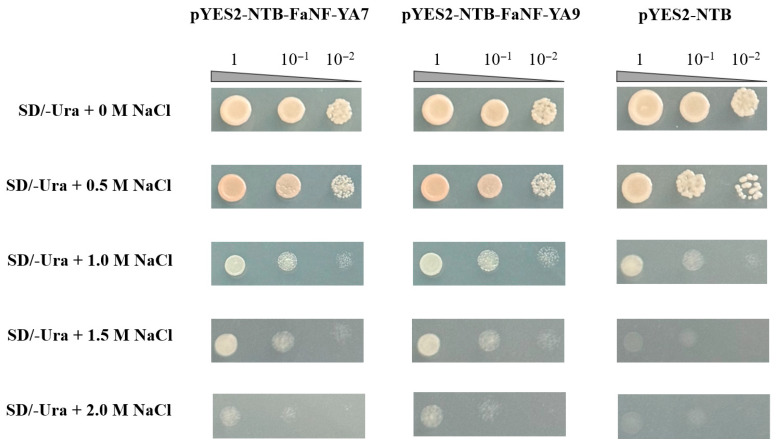
Heterologous expression of FaNF-YA7 and FaNF-YA9 enhances salt tolerance in yeast. pYES2-NTB is the negative control. SD means nutrition-deprived yeast medium; 1, 10^−1^, 10^−2^ indicate different dilutions of yeast fluids.

To investigate whether FaNF-YA7 and FaNF-YA9 play functional roles in salt stress response, they were cloned into the pYES2-NTB vector, respectively. The recombinant constructs (pYES2-NTB-FaNF-YA7 and pYES2-NTB-FaNF-YA9) and the empty vector (negative control) were transformed into a salt-sensitive yeast strain. As shown in Figure 12, the negative control formed visible colonies on SD/-Ura medium containing 0, 0.5, and 1.0 M NaCl, but failed to produce any detectable growth at 1.5 M and 2.0 M NaCl. In contrast, yeast cells expressing FaNF-YA7 or FaNF-YA9, although also showing reduced growth at higher salt concentrations, still exhibited discernible growth traces on plates containing 1.5 M and 2.0 M NaCl. This suggested potential biological functions of FaNF-YA7 and FaNF-YA9 in salt stress response.

## 3. Discussion

Unlike animals and yeast, where each NF-Y subunit is encoded by a single gene, plants have undergone extensive expansion of the NF-Y gene family through gene duplication events [9]. In the present study, we identified 27 *FaNF-YA* genes in the octoploid cultivated strawberry (*Fragaria* × *ananassa*). This number is substantially larger than the six NF-YA members reported in the diploid woodland strawberry (*Fragaria vesca*), which is consistent with the allo-octoploid genome structure of cultivated strawberry [4]. Phylogenetic analysis revealed that the 27 FaNF-YA proteins were clearly divided into four distinct groups (Groups 1–4), and this grouping was strongly supported by conserved motif composition and gene structure analysis. Notably, the results of gene structure analysis showed that Group 2 members (*FaNF-YA6–FaNF-YA13*) exhibited relatively simple exon–intron structures, with several genes being intron-less. In contrast, Group 1 members possessed more complex intron–exon patterns. This structural divergence may reflect different evolutionary constraints and functional trajectories among subgroups. The intraspecies collinearity analysis identified numerous duplicated *FaNF-YA* gene pairs, and the calculated Ka/Ks ratios revealed that the vast majority of these pairs exhibited Ka/Ks values below 1, which indicated that purifying selection had been the dominant evolutionary force acting on this gene family. This is consistent with the notion that the core DNA-binding and subunit interaction functions of NF-YA proteins are under strong functional constraints. Only two gene pairs (*FaNF-YA1/FaNF-YA2* and *FaNF-YA24/FaNF-YA26*) showed Ka/Ks ratios exceeding 1, which suggested positive selection might have driven functional divergence in these specific members. These findings are in line with the observation that the non-conserved N- and C-termini of NF-YA proteins are under pressure for diversifying selection, which may provide clues to functional diversification following gene duplication. To investigate the biological processes that *FaNF-YA* were probably involved in, we performed *cis*-acting element analysis of *FaNF-YA* promoters. The promoters of *FaNF-YA* genes harbored abundant stress-related and phytohormone-responsive *cis*-acting elements, including ABRE (ABA-responsive element), MYB and MYC binding sites, MeJA-responsive elements, and DRE (dehydration-responsive element).

A key finding of this study is the pronounced organ-specific and stress-responsive expression divergence among FaNF-YA members. Organ-specific expression profiling revealed that different *FaNF-YA* genes exhibited preferential expression in distinct organs. For example, *FaNF-YA9/11/12* were predominantly expressed in leaves, stems, and flowers, with exceptionally high transcript abundance, while *FaNF-YA1* was relatively evenly expressed across most organs. In contrast, *FaNF-YA3* and *FaNF-YA5* were expressed at very low levels across all examined organs. All *FaNF-YA* genes or groups showed extremely low expression in fruit, with fruit/root ratios ranging from 0.01 to 0.04. This suggests that FaNF-YA family members may play limited roles during fruit development under normal growth conditions. This observation contrasts with findings in tomato and banana, where certain NF-Y genes have been implicated in fruit ripening regulation [44]. Under salt stress, FaNF-YA members exhibited diverse temporal expression patterns, which reflected distinct regulatory roles. Notably, the groups *FaNF-YA14/16/18/22*, *FaNF-YA7/8/10/13*, and *FaNF-YA9/11/12* were most strongly and rapidly induced, peaking at 12 h post-treatment, with fold changes reaching as high as 230- to 360-fold. In contrast, *FaNF-YA3* showed essentially no response to salt stress. This salt-induced upregulation of specific FaNF-YA members is consistent with previous studies in other plant species. In *Arabidopsis*, the NF-YA transcription factor family has been shown to play a critical role in response to high salt concentrations and other abiotic stresses [45]. In soybean, *GmNFYA5* exhibits the highest transcript induction among all NF-YA family members under drought stress, and its overexpression enhances drought tolerance via both ABA-dependent and ABA-independent pathways [24]. Our findings collectively indicate that specific FaNF-YA members are promising candidate genes for improving salt tolerance in strawberry through molecular breeding approaches.

To functionally validate the involvement of *FaNF-YA* genes in salt stress responses, we performed heterologous expression assays in yeast. Yeast cells expressing FaNF-YA7 or FaNF-YA9 exhibited enhanced salt tolerance. Although the growth of all transformants decreased with increasing salt concentrations, the FaNF-YA7- and FaNF-YA9-expressing yeast strains consistently showed discernible growth at 1.5 M and 2.0 M NaCl, where the negative control was completely inhibited. This heterologous functional assay provides direct evidence that FaNF-YA7 and FaNF-YA9 possess intrinsic capacity to enhance salt tolerance. Given that yeast lacks the NF-YB and NF-YC subunits that typically form the heterotrimeric complex with NF-YA in plants, the observed salt tolerance effect in yeast may occur through alternative mechanisms. Furthermore, both FaNF-YA7 and FaNF-YA9 proteins failed to activate reporter gene expression in the yeast system, in contrast to the positive control. This suggests that FaNF-YA7 and FaNF-YA9 may not function as autonomous transcriptional activators but instead require interaction with NF-YB and NF-YC subunits to form the functional heterotrimeric complex necessary for transactivation. This is consistent with the established paradigm of NF-Y function, where the NF-YA subunit provides DNA-binding specificity, while transcriptional activation is often mediated through the NF-YC subunit or through recruitment of co-activators.

Despite the comprehensive genome-wide identification and characterization of the FaNF-YA gene family in this study, several important questions remain to be addressed. First, while we observed that specific FaNF-YA members were dramatically induced by salt stress, the downstream target genes they regulate remain unknown. Future studies employing chromatin immunoprecipitation sequencing (ChIP-seq) or transcriptomic analysis of transgenic lines overexpressing or silencing *FaNF-YA* genes could identify direct targets and elucidate the transcriptional regulatory networks. Second, the functional roles of *FaNF-YA* genes in other abiotic stresses, such as drought, cold, and heat stress, warrant investigation. Given the presence of multiple stress-related *cis*-elements (e.g., LTR for cold, DRE for dehydration) in their promoters, it is likely that *FaNF-YA* genes are involved in responses to a broader range of environmental stresses. NF-Y has been recognized as a key regulator in plant responses to a wide range of adverse stresses, including high salinity, drought, extreme temperatures, nutrient deficiency, hypoxia, and heavy metal toxicity [44]. Third, stable genetic transformation of strawberry to overexpress or knock out specific *FaNF-YA* genes (particularly the highly salt-responsive members, such as those in the *FaNF-YA14/16/18/22* group) is needed to confirm their functional roles in planta and to assess their potential for improving stress tolerance in strawberry cultivation. Advances in strawberry transformation and genome editing technologies make such approaches increasingly feasible.

## 4. Materials and Methods

### 4.1. Identification and Physicochemical Characterization of NF-YA Gene Family in Strawberry

Genomic resources for cultivated strawberry (*Fragaria* × *ananassa*), including genome assembly, coding sequences (CDS), and protein annotations, were retrieved from the Phytozome Database (https://phytozome-next.jgi.doe.gov/info/Fxananassa_v1_0_a1, accessed on 17 January 2026). Two complementary strategies were employed to identify *FaNF-YA* genes in the strawberry genome. First, the amino acid sequences of 10 *AtNF-YA* genes from *Arabidopsis thaliana* (AT1G17590, AT1G30500, AT1G54160, AT1G72830, AT2G34720, AT3G05690, AT3G14020, AT3G20910, AT5G06510, AT5G12840) were downloaded from the TAIR database (https://phytozome-next.jgi.doe.gov/info/Athaliana_TAIR10, accessed on 18 January 2026). These sequences were used as queries to perform BLASTP [46] searches against the strawberry genome with an E-value cutoff of 1 × 10^−5^, a minimum query coverage of 50%, and a minimum sequence identity of 40%. Second, the Hidden Markov Model (HMM) profile corresponding to the NF-YA conserved domain (PF02045) was retrieved from the InterPro database (https://www.ebi.ac.uk/interpro/, accessed on 19 January 2026). The HMMER v3.1 tool was then employed to scan the strawberry protein database using the PF02045 profile, with an E-value threshold of 0.01 to identify additional putative NF-YA family members. All candidate sequences obtained from both methods were manually inspected and validated by confirming the presence of the complete NF-YA conserved domain using the NCBI Conserved Domain Database (CDD, https://www.ncbi.nlm.nih.gov/Structure/cdd/, accessed on 19 January 2026) and the SMART tool (http://smart.embl-heidelberg.de/, accessed on 19 January 2026). Redundant and incomplete sequences were removed.

The physicochemical properties of the identified FaNF-YA proteins, including molecular weight (kDa), theoretical isoelectric point (pI), instability index, aliphatic index, and grand average of hydropathicity (GRAVY), were analyzed using the ExPASy ProtParam tool (https://web.expasy.org/protparam/, accessed on 21 January 2026).

### 4.2. Multiple Sequence Alignment and Phylogenetic Analysis

Multiple sequence alignment of the full-length FaNF-YA protein sequences was performed using the MUSCLE algorithm implemented in TBtools (v2.441) [47] with default parameters. To examine the evolutionary relationships of FaNF-YA proteins, NF-YA sequences from *Arabidopsis thaliana* (10 AtNF-YAs), *Fragaria vesca* (6 FvNF-YAs), and *Malus domestica* (11 MdNF-YAs) were retrieved from the Phytozome database.

A phylogenetic tree was constructed using the neighbor-joining (NJ) method [48] with 1000 bootstrap replicates in TBtools (v2.441).

### 4.3. Gene Structure and Conserved Motif Analysis

The exon–intron architectures of *FaNF-YA* genes were visualized using the Gene Structure Display Server 2.0 (GSDS 2.0, https://gsds.gao-lab.org/, accessed on 25 January 2026) [49] by comparing the coding sequences (CDS) with the corresponding genomic sequences retrieved from the strawberry genome database.

Conserved protein motifs in FaNF-YA sequences were identified using the MEME Suite (http://meme-suite.org/, accessed on 27 January 2026) [50] with default parameters. The distribution of identified motifs among FaNF-YA members was visualized using TBtools (v2.441).

### 4.4. Intraspecies and Interspecies Collinearity Analysis and Ka/Ks Calculation

The Gff3 (General Feature Format 3) annotation files and genome sequence files for cultivated strawberry (*Fragaria* × *ananassa*), diploid strawberry (*Fragaria vesca*), *Arabidopsis* (*Arabidopsis thaliana*) and apple (*Malus domestica*) were retrieved from the Phytozome database. Intraspecies and interspecies collinearity analyses were performed and visualized using TBtools (v2.441) with the MCScanX algorithm under default parameters.

To estimate the selection pressure acting on duplicated *FaNF-YA* gene pairs, the non-synonymous (Ka) and synonymous (Ks) substitution rates were calculated using the KaKs_Calculator embedded in TBtools. The Ka/Ks ratio was used to infer the type of selection: Ka/Ks < 1 indicates purifying (stabilizing) selection, Ka/Ks = 1 indicates neutral evolution, and Ka/Ks > 1 indicates positive (Darwinian) selection.

### 4.5. cis-Acting Element Analysis

The 2000 bp promoter sequences upstream of the translation start codon of each *FaNF-YA* gene were retrieved from the Phytozome database. These sequences were analyzed for the presence of putative *cis*-acting regulatory elements using the online tool PlantCARE (https://bioinformatics.psb.ugent.be/webtools/plantcare/html/, accessed on 5 February 2026). The identified *cis*-elements were categorized based on their predicted functions (e.g., stress responsiveness, phytohormone responsiveness, light responsiveness, and plant growth/development). The distribution of *cis*-elements across the *FaNF-YA* promoters was visualized using TBtools (v2.441).

### 4.6. Plant Materials and Treatments

Strawberry (*Fragaria* × *ananassa*) seedlings used in this study were maintained at the School of Agriculture, Liaodong University (Dandong, China). After establishment in the field for 6–8 weeks, healthy strawberry plants at the vegetative growth stage (with approximately 6–8 fully expanded leaves and a well-developed root system) were used for root, stem, and leaf sampling. For stolon and stolon leaf collection, plants were further grown under long-day conditions (16 h light/8 h dark) for an additional 4–6 weeks until stolons emerged. Flowers were collected at the visible bud to full bloom stage (approximately 10–12 weeks after planting). Fruits were harvested at the commercial mature stage (red color development, approximately 30–35 days after anthesis).

Strawberry (*Fragaria* × *ananassa* cv. ‘Red Face’) seedlings were cultivated in a controlled-environment greenhouse with the following conditions: day/night temperature of 25 °C ± 2 °C/18 °C ± 2 °C, relative humidity of 60% ± 5%, photosynthetic photon flux density (PPFD) of 150 μmol·m^−2^·s^−1^ and a photoperiod of 16 h light/8 h dark. After field planting for 6–8 weeks (to ensure establishment of a well-developed root system), healthy and uniformly sized seedlings were selected based on the following criteria: presence of 6–8 fully expanded true leaves, absence of visible disease or pest damage. For stress treatments, only seedlings meeting all these criteria were used. For organ-specific expression profiling, additional organs (stolon, flower, fruit) were collected at their respective optimal developmental stages. The relative water content (RWC) was measured and calculated as González et al.’s method [51].

To prevent confounding effects from repeated sampling, an independent sampling design was used: for each salt stress time point (3, 12, 24, and 48 h), a distinct set of plants was subjected to 200 mM NaCl treatment and harvested at that specific time point. A separate cohort of control plants (0 h) was similarly maintained under identical conditions without salt treatment. Each treatment–time combination had three independent biological replicates (each replicate consisting of pooled leaf tissue from three individual plants). A single, common control group of untreated plants (0 h) was used for comparison with all stress treatments. For salt stress treatment, strawberry seedlings were subjected to NaCl (200 mM) in hydroponic culture. The 3 h, 12 h, 24 h, and 48 h time points post-treatment were selected to capture the dynamic transcriptional profiles of the stress response. The samples were immediately frozen in liquid nitrogen and stored at −80 °C. Three biological replicates were performed for each treatment and time point.

### 4.7. qRT-PCR and Expression Analysis

Total RNA was extracted from strawberry leaf samples using an RNA extraction kit (Bioteke, Beijing, China) following the manufacturer’s instructions. RNA integrity was verified by 1% agarose gel electrophoresis. The extracted RNA was reverse-transcribed into single-stranded cDNA using a reverse transcription kit (PrimeScript™ RT Reagent Kit, Takara Bio, Kusatsu, Japan). The obtained cDNA was diluted 10-fold to serve as the template for qRT-PCR analysis.

qRT-PCR primers were designed using Primer 5.0 (Premier Biosoft, Palo Alto, CA, USA) based on the full-length coding sequences of *FaNF-YA* genes. The *FaActin* gene was selected as the internal reference control [52]. Three biological replicates and three technical replicates were used for each sample. qRT-PCR was performed using the THUNDERBIRD Next SYBR qPCR Mix (TOYOBO, Osaka, Japan) according to the manufacturer’s protocol. The 20 µL reaction mixture was subjected to the following thermal cycling protocol: initial denaturation at 95 °C for 5 min, followed by 45 cycles of denaturation at 95 °C for 15 s and annealing/extension at 60 °C for 5 min. The reaction was carried out on an Applied Biosystems 7500 Fast Real-Time PCR System (Waltham, MA, USA). All reactions were repeated three times, and the relative gene expression levels were calculated using the 2^−ΔΔCt^ method [53]. Normalized count values were log_2_-transformed to stabilize variance and better meet the assumptions of normality underlying the statistical models used. Differences in significance were analyzed with Duncan’s multiple range test (*p* < 0.05).

Due to extremely high sequence similarity among certain *FaNF-YA* genes, conventional primer design could not achieve gene-specific amplification. Therefore, a single primer pair was designed to co-amplify each combination of *FaNF-YA* genes with high sequence similarity. The combinations included: *FaNF-YA7*/*8*/*10*/*13*, *FaNF-YA9*/*11*/*12*, *FaNF-YA14*/*16*/*18*/*22*, *FaNF-YA15*/*17*/*19*/*20*/*11*, and *FaNF-YA6*/*23*/*24*/*25*/*26*/*27*. The heatmaps were constructed using TBtools (v2.441).

### 4.8. Transcription-Activating Activity Verification

To assess the transcriptional activation activity of FaNF-YA proteins, FaNF-YA7 and FaNF-YA9 were selected as representative members. The full-length coding sequences of *FaNF-YA7* and *FaNF-YA9* were amplified and cloned into the pGBKT7 yeast expression vector, which contains the GAL4 DNA-binding domain. The recombinant vectors pGBKT7-FaNF-YA7 and pGBKT7-FaNF-YA9, along with pGBKT-53/pGADT7-T (positive control) and empty pGBKT7 (negative control), were transformed into yeast competent cells (Y2H strain, Takara Bio, Kusatsu, Japan), respectively.

Transformants were first selected on SD/-Trp medium (synthetic dropout medium lacking tryptophan) to confirm successful transformation. After verification of normal growth on SD/-Trp, the yeast cultures were spotted onto nutrition-deprived medium SD/-Trp/-Ade/-His (lacking tryptophan, adenine, and histidine) supplemented with X-α-Gal (40 mg/L) and Aureobasidin A (AbA, 500 ng/L). The inclusion of X-α-Gal and AbA provided more detectable and reliable evidence for the activation of the yeast GAL4 system. Plates were incubated at 30 °C for 3–5 days, and colony growth and blue coloration were recorded as indicators of transcriptional activation activity.

### 4.9. Salt-Resistant Yeast Transformation

To investigate the potential role of FaNF-YA proteins in salt stress response, FaNF-YA7 and FaNF-YA9 were selected as representatives. The full-length coding sequences of *FaNF-YA7* and *FaNF-YA9* were amplified and cloned into the pYES2-NTB vector, respectively. pYES2-NTB-FaNF-YA7, pYES2-NTB-FaNF-YA9 and negative control (pYES2-NTB blank vector) were transformed into salt-resistant yeast (INVSc1), respectively. The transformed yeast was cultured on nutrition-deprived media (SD/-Ura) containing different concentrations of salt (NaCl). The concentrations of salt were 0, 0.5, 1.0, 1.5 and 2.0 M. The growth state of yeast on the media indicated salt tolerance.

## 5. Conclusions

This study provides a comprehensive genome-wide analysis of the NF-YA gene family in cultivated strawberry, identifying 27 FaNF-YA members and characterizing their phylogenetic relationships, gene structures, conserved motifs, promoter *cis*-acting elements, expression patterns under salt stress, and functional roles in yeast. These results reveal that FaNF-YA members have evolved diverse expression patterns and regulatory mechanisms, with specific members being strongly induced by salt stress and conferring enhanced salt tolerance in a heterologous system. These findings establish a solid foundation for future functional studies aimed at elucidating the molecular mechanisms by which FaNF-YA transcription factors regulate stress responses in strawberry and provide candidate genes for molecular breeding of stress-tolerant strawberry cultivars.

## Figures and Tables

**Table 1:** Physiochemical characteristics of FaNF-YAs.

Gene Name	Gene ID	Molecular Weight (Da)	Isoelectric Points	Amino Acid No.	Instability Index	Aliphatic Index	GRAVY
FaNF-YA1	maker-Fvb1-1-snap-gene-238.58-mRNA-1	40,630.64	9.07	366	57.04	59.67	−0.657
FaNF-YA2	maker-Fvb1-2-snap-gene-7.45-mRNA-1	39,806.51	9.14	359	54.59	58.38	−0.702
FaNF-YA3	maker-Fvb1-2-snap-gene-58.63-mRNA-1	43,671.39	9.2	392	53.26	67.63	−0.533
FaNF-YA4	maker-Fvb1-3-augustus-gene-39.58-mRNA-1	33,928.88	9.24	309	53.16	57.73	−0.746
FaNF-YA5	maker-Fvb1-4-snap-gene-35.77-mRNA-1	46,996.26	8.47	424	50.91	72.85	−0.411
FaNF-YA6	snap_masked-Fvb3-1-processed-gene-116.32-mRNA-1	20,458.73	10.13	185	58.61	50.76	−1.084
FaNF-YA7	maker-Fvb3-1-augustus-gene-265.34-mRNA-1	22,455.78	6.79	204	71.46	54.12	−0.959
FaNF-YA8	maker-Fvb3-2-augustus-gene-53.31-mRNA-1	22,519.86	6.79	204	69.78	53.63	−0.929
FaNF-YA9	maker-Fvb3-2-augustus-gene-192.30-mRNA-1	37,194.68	7.26	340	53.02	49.97	−1.011
FaNF-YA10	augustus_masked-Fvb3-3-processed-gene-38.9-mRNA-1	18,611.57	7.9	170	70.21	59.71	−0.94
FaNF-YA11	maker-Fvb3-3-augustus-gene-171.42-mRNA-1	37,223.83	8.32	340	55.04	49.97	−0.999
FaNF-YA12	maker-Fvb3-4-augustus-gene-118.22-mRNA-1	37,290.84	8.31	340	54.04	50.53	−1.01
FaNF-YA13	augustus_masked-Fvb3-4-processed-gene-239.4-mRNA-1	22,475.76	6.79	204	70.72	53.63	−0.967
FaNF-YA14	maker-Fvb6-1-augustus-gene-122.23-mRNA-1	31,969.84	9.53	289	47.51	62.73	−0.674
FaNF-YA15	maker-Fvb6-1-augustus-gene-222.26-mRNA-1	32,293.23	9.77	293	45.45	55.22	−0.764
FaNF-YA16	maker-Fvb6-2-snap-gene-27.59-mRNA-1	32,014.97	9.46	290	46.89	65.55	−0.605
FaNF-YA17	maker-Fvb6-2-augustus-gene-138.31-mRNA-1	31,881.82	9.87	291	45.31	59.62	−0.683
FaNF-YA18	maker-Fvb6-3-augustus-gene-117.26-mRNA-1	32,505.57	9.51	294	45.19	66.63	−0.609
FaNF-YA19	maker-Fvb6-3-augustus-gene-233.32-mRNA-1	32,209.09	9.82	293	46.81	56.55	−0.737
FaNF-YA20	maker-Fvb6-3-augustus-gene-252.26-mRNA-1	32,220.11	9.87	293	48.56	56.21	−0.738
FaNF-YA21	maker-Fvb6-4-augustus-gene-147.20-mRNA-1	32,328.21	9.79	293	49.51	56.55	−0.749
FaNF-YA22	snap_masked-Fvb6-4-processed-gene-256.15-mRNA-1	28,725.41	9.69	259	40.89	66.25	−0.601
FaNF-YA23	maker-Fvb7-1-augustus-gene-6.20-mRNA-1	36,395.58	9.4	333	40.2	56.85	−0.757
FaNF-YA24	maker-Fvb7-2-augustus-gene-9.40-mRNA-1	36,640.84	9.52	333	39.84	56.82	−0.78
FaNF-YA25	maker-Fvb7-3-augustus-gene-240.23-mRNA-1	36,346.41	9.48	333	37.08	55.68	−0.784
FaNF-YA26	maker-Fvb7-4-augustus-gene-214.32-mRNA-1	36,559.64	9.61	333	38.87	53.93	−0.808
FaNF-YA27	snap_masked-Fvb7-4-processed-gene-217.11-mRNA-1	15,868.69	9.67	145	26.82	53.79	−0.669

**Table 2:** Non-synonymous (Ka) and synonymous (Ks) substitution rates of duplicated *FaNF-YA* gene pairs in *Fragaria* × *ananassa*.

Duplicated Gene Pairs	Ka	Ks	Ka/Ks	Effective Length
FaNF-YA1/FaNF-YA2	0.020207366	0.018262654	1.106485712	1077
FaNF-YA1/FaNF-YA3	0.022266203	0.030034043	0.741365474	1098
FaNF-YA1/FaNF-YA4	0.012745173	0.02364262	0.539076192	927
FaNF-YA1/FaNF-YA5	0.03126401	0.062557597	0.499763611	1095
FaNF-YA1/FaNF-YA14	0.400544954	1.349672283	0.296772008	762
FaNF-YA1/FaNF-YA16	0.428111786	1.328641474	0.322217689	807
FaNF-YA1/FaNF-YA18	0.399976218	1.552689837	0.257602135	792
FaNF-YA1/FaNF-YA22	0.414959977	1.578681111	0.26285231	723
FaNF-YA2/FaNF-YA3	0.017110427	0.008061885	2.122385495	1077
FaNF-YA3/FaNF-YA4	0.01418482	0.018809763	0.754120068	927
FaNF-YA2/FaNF-YA4	0.017773344	0.021194503	0.838582702	927
FaNF-YA3/FaNF-YA5	0.03024794	0.066214768	0.456815621	1173
FaNF-YA2/FaNF-YA5	0.024036568	0.043281238	0.555357685	1074
FaNF-YA2/FaNF-YA14	0.409427071	1.259190945	0.325150901	765
FaNF-YA3/FaNF-YA14	0.401083427	1.288817736	0.311202597	768
FaNF-YA2/FaNF-YA16	0.436329555	1.250158916	0.349019272	810
FaNF-YA3/FaNF-YA16	0.431048265	1.278185466	0.337234523	813
FaNF-YA2/FaNF-YA18	0.411849318	1.434552915	0.287092455	795
FaNF-YA3/FaNF-YA18	0.40049711	1.473641722	0.271773732	798
FaNF-YA3/FaNF-YA22	0.415454462	1.531795878	0.271220512	729
FaNF-YA2/FaNF-YA22	0.424409792	1.493596934	0.284152828	726
FaNF-YA4/FaNF-YA5	0.015673807	0.048054864	0.326164842	924
FaNF-YA4/FaNF-YA14	0.408405251	1.261931926	0.32363493	762
FaNF-YA4/FaNF-YA16	0.429865276	1.249966402	0.343901464	807
FaNF-YA4/FaNF-YA18	0.403106375	1.46537978	0.275086623	792
FaNF-YA4/FaNF-YA22	0.420223224	1.496495488	0.280804872	723
FaNF-YA5/FaNF-YA14	0.389203127	1.254300953	0.310294851	762
FaNF-YA5/FaNF-YA16	0.408273665	1.218503879	0.335061441	807
FaNF-YA5/FaNF-YA18	0.392105115	1.495089551	0.26226196	792
FaNF-YA5/FaNF-YA22	0.40590254	1.486538058	0.273052235	723
FaNF-YA7/FaNF-YA8	0.008683165	0.034409704	0.2523464	612
FaNF-YA6/FaNF-YA9	0.007172798	0.030288027	0.236819591	555
FaNF-YA6/FaNF-YA11	0.004782798	0.007416624	0.644875368	555
FaNF-YA7/FaNF-YA10	0.005156875	0.033856242	0.152316807	510
FaNF-YA7/FaNF-YA13	0.004335272	0.048403891	0.089564539	612
FaNF-YA8/FaNF-YA10	0.00773535	0.008368288	0.924364741	510
FaNF-YA9/FaNF-YA11	0.011736819	0.02467666	0.475624266	1020
FaNF-YA8/FaNF-YA13	0.004329016	0.027400307	0.157991524	612
FaNF-YA9/FaNF-YA12	0.014356066	0.046023499	0.31192905	1020
FaNF-YA10/FaNF-YA13	0.002574005	0.016737096	0.153790422	510
FaNF-YA11/FaNF-YA12	0.01305658	0.028839804	0.452727773	1020
FaNF-YA14/FaNF-YA16	0.015562742	0.03481769	0.446978027	855
FaNF-YA15/FaNF-YA17	0.013647079	0.034517184	0.395370564	873
FaNF-YA14/FaNF-YA18	0.013835904	0.019247247	0.718851035	867
FaNF-YA15/FaNF-YA20	0.016566939	0.034488835	0.480356576	879
FaNF-YA15/FaNF-YA19	0.010494924	0.044715227	0.234705815	879
FaNF-YA15/FaNF-YA21	0.015030563	0.039678674	0.378807096	879
FaNF-YA14/FaNF-YA22	0.006824045	0.016115213	0.4234536	777
FaNF-YA16/FaNF-YA18	0.013742591	0.039272775	0.349926658	870
FaNF-YA17/FaNF-YA20	0.01060356	0.019465813	0.544727296	873
FaNF-YA17/FaNF-YA19	0.004523763	0.029439593	0.153662566	873
FaNF-YA16/FaNF-YA22	0.013903367	0.038987896	0.356607268	765
FaNF-YA19/FaNF-YA20	0.005988056	0.009661969	0.6197552	879
FaNF-YA20/FaNF-YA21	0.007488829	0.024432128	0.306515631	879
FaNF-YA19/FaNF-YA21	0.004482085	0.034488835	0.129957566	879
FaNF-YA18/FaNF-YA22	0.01284278	0.029841188	0.430370927	777
FaNF-YA23/FaNF-YA24	0.022308916	0.022498875	0.991556978	999
FaNF-YA23/FaNF-YA25	0.010438355	0.036056511	0.289499858	999
FaNF-YA23/FaNF-YA26	0.022313797	0.031666973	0.704639414	999
FaNF-YA24/FaNF-YA25	0.015686846	0.022456767	0.69853537	999
FaNF-YA24/FaNF-YA26	0.030300498	0.018032424	1.680334191	999
FaNF-YA25/FaNF-YA26	0.017012725	0.022439969	0.758143891	999

## Data Availability

Data is contained within the article and Supplementary Materials.

## Supplementary Materials

The following supporting information can be downloaded at: https://www.mdpi.com/article/10.3390/plants15101475/s1. Table S1: Collinear *NF-YA* gene pairs in between *Fragaria* × *ananassa* and *Arabidopsis thaliana*. Table S2: Collinear *NF-YA* gene pairs in between *Fragaria* × *ananassa* and *Fragaria vesca*. Table S3: Collinear *NF-YA* gene pairs in between *Fragaria* × *ananassa* and *Malus domestica*. Table S4a: Analysis of *cis*-acting elements in *FaNF-YA* promoters. Table S4b: Number of *cis*-acting elements in *FaNF-YAs* promoters. Table S5: qRT-PCR data of FaNF-YA tissue-specific expression pattern. Table S6: qRT-PCR data of *FaNF-YA*s expression pattern under salt treatment. Table S7: Primer sequences for the quantification of *FaNF-YAs*.
